# Intestinal and Urinary Pseudomyiasis by Psychodinae Larvae in an Adolescent: A Fact, Fallacy, or Harmless Spectator?

**DOI:** 10.7759/cureus.77482

**Published:** 2025-01-15

**Authors:** Maria M Resende, Joana Caniço, Maria M Flores

**Affiliations:** 1 Department of Pediatrics, Unidade Local de Saúde da Região de Aveiro, Hospital Infante D. Pedro, Aveiro, PRT; 2 Department of Clinical Pathology, Unidade Local de Saúde da Região de Aveiro, Hospital Infante D. Pedro, Aveiro, PRT

**Keywords:** clogmia albipunctata, drain fly, myiasis, pseudomyiasis, psychodinae larvae

## Abstract

Myiasis is caused by fly larvae and can be classified into obligatory, facultative, and accidental (or pseudomyiasis) forms. *Clogmia albipunctata*, a species of *Psychodinae* fly, commonly found in areas such as kitchens, toilets, and drains, has been linked to urogenital and intestinal myiasis. However, recent evidence questions its role as a true causative agent of myiasis. We report a 17-year-old female who experienced intermittent larval elimination in her stool and urine, accompanied by gastrointestinal symptoms. *Psychodinae* larvae, likely* Clogmia *spp., were identified in both stool and urine samples collected from her home. No other relevant findings were found during the investigation. After explaining the fly's life cycle to the adolescent and her parents, they recognized the presence of a similar fly near the toilet. This led to a renovation of the sanitation facilities and isolation of the drainage system, after which larval elimination ceased with no recurrence. The breathing requirements of* Psychodinae* larvae suggest that it is unlikely they could survive, let alone develop, in the gastrointestinal or urinary tracts. Additionally, their presence has not been documented in these areas, and the suspected causative agent was not directly observed in the patient. In this case, as with similar reports, definitive conclusions are difficult to reach regarding whether these instances represent pseudomyiasis or are merely incidental findings in the environment. However, the evidence tends to favor the latter hypothesis and rules out the possibility of true myiasis. This report highlights the importance of accurately identifying the implicated species and eliminating the source of infestation to prevent recurrence.

## Introduction

Myiasis is a disease caused by fly larvae and is classified into three types: obligatory, facultative, and pseudomyiasis. The first two are types of true myiasis [1]. In obligatory myiasis, fly larvae require healthy tissue from a living host for their development, leading to tissue destruction and inflammation. In facultative myiasis, larvae opportunistically feed on wounded or necrotic tissue following oviposition or larviposition. In contrast, pseudomyiasis, or accidental myiasis, refers to the incidental infestation of the human body with fly larvae that do not rely on host tissue as a food source [1-3].

This condition can also be categorized based on the affected tissue. Cutaneous myiasis is the most common type in humans. Cavitary forms, including ophthalmic, ear-nose-throat, gastrointestinal, and urogenital, among others, are less common [1,3]. Myiasis is predominantly caused by fly larvae from the suborder Brachycera [1,4]. However, *Psychodinae* fly larvae (family *Psychodidae*, suborder Nematocera, order Diptera) have also been reported worldwide as causative agents. Among these, *Clogmia* (*Telmatoscopus*) spp., particularly *Clogmia albipunctata*, is one of the most commonly implicated species, primarily associated with urogenital, intestinal, and nasopharyngeal myiasis [2,3]. They are cosmopolitan species found worldwide, particularly in tropical and subtropical regions, with low socioeconomic status and poor hygienic and sanitation conditions [2,3]. 

*C. albipunctata*, called the drain fly or sewer fly due to its association with these types of microhabitats, is commonly found in areas such as kitchens, toilets, showers, and floor drains, where sufficient organic material may accumulate and is accessible to adult flies for oviposition. Due to their low flying abilities, they are often found near their breeding sites. However, larvae develop in hidden locations, such as plumbing systems, feeding on bacteria and biofilms formed from human waste and decaying organic matter [2,3].

These non-biting flies are generally regarded as having minimal impact on human health, aside from their potential roles as mechanical vectors for bacteria or allergens. However, as their larval stages have been implicated in cases of myiasis, recent evidence has emerged, highlighting growing concerns about their role in this condition and the associated challenges in its investigation and diagnosis [2-4].

This article aims to report a case initially suspected to be urinary and intestinal pseudomyiasis in an adolescent, followed by a critical discussion of the investigative process and the necessary interventions.

This study was previously presented as a poster at the 21st National Paediatrics Congress of the Portuguese Society of Paediatrics in 2021 and at the 11^th^ National Clinical Pathology Congress of the Portuguese Society of Clinical Pathology in 2022.

## Case presentation

We present a case of a 17-year-old female adolescent referred to a pediatric consultation with complaints of abdominal discomfort and reporting the passage of worm-like organisms in her stools and urine. She was healthy and had no cognitive deficits. The adolescent lived in a rural area of Portugal, came from a favorable social background, and had no recent history of travel abroad.

Four months prior to the first pediatric consultation, she experienced abdominal pain, bloating, constipation, frequent eructation, nausea, asthenia, and loss of appetite. She reported observing "elongated white worms" in her stools and was diagnosed by her family doctor with parasitosis, caused by *Ascaris lumbricoides*. Despite requiring multiple courses of anthelmintics and a laxative, her parasitic infection resolved, with significant improvement in her gastrointestinal symptoms.

Two months later, despite the overall improvement in her gastrointestinal symptoms, she still experienced occasional abdominal discomfort, associated with asthenia and loss of appetite. At that point, she began to notice intermittent elimination of worm-like organisms in her stools and later also in her urine, both of which were observed in the toilet. Importantly, she never reported any genitourinary symptoms and did not feel or observe anything unusual in the perianal or genital regions, nor in her underwear. The worm-like organisms were distinctly different from the previously expelled parasite, varying in size from smaller, whitish forms to larger, brownish ones. They were eliminated every 15-20 days over periods of two to four days, approximately numbering five to 10, with some still alive.

**Figure 1:**
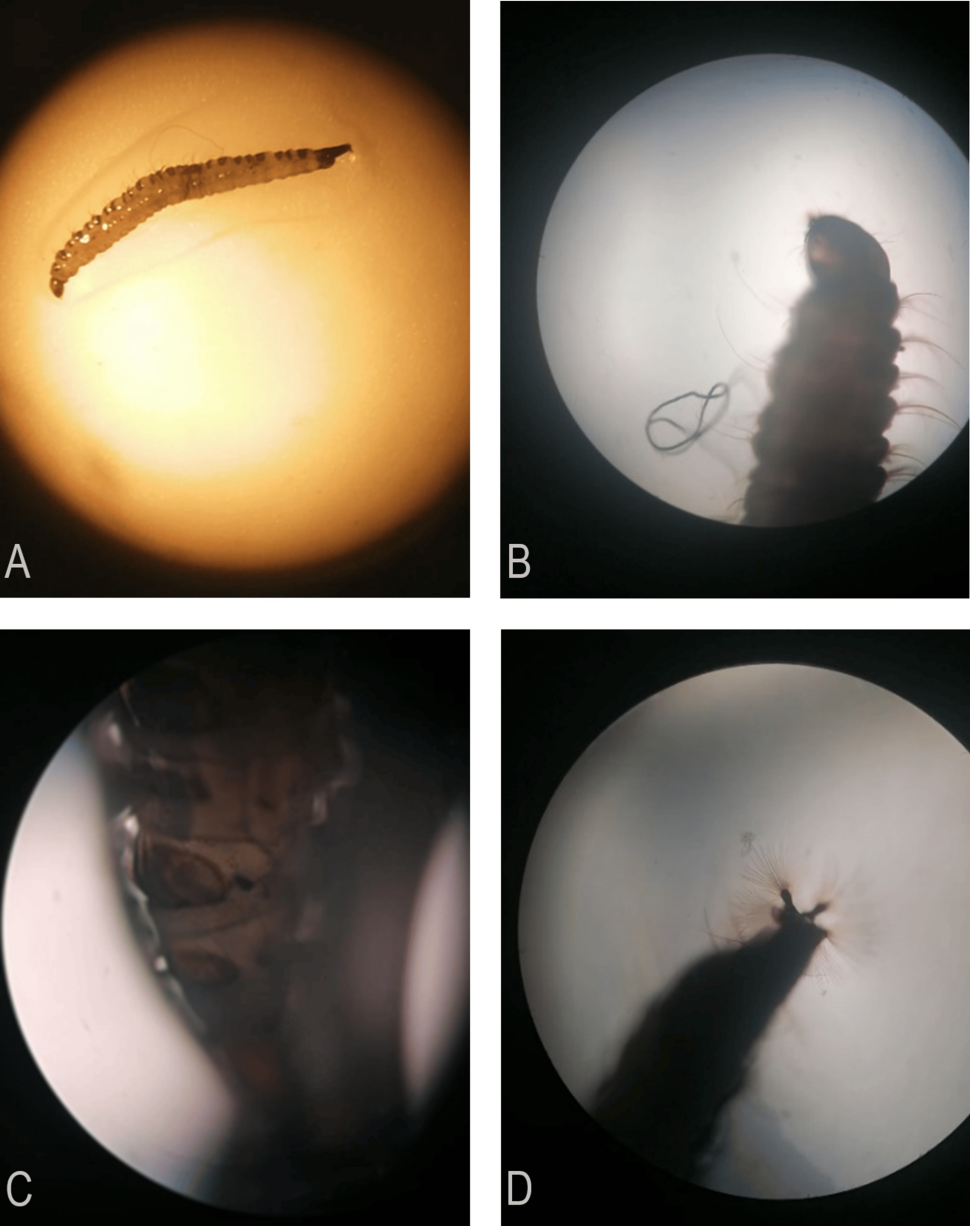
Morphological examination of Psychodinae larvae using manual optical microscopy techniques by the local hospital's microbiology team. (A) Lateral view of 10-mm brownish larvae with a lighter ventral surface, a darker dorsal surface, and even darker extremities. (B) Lateral view of the head. (C) Lateral view of body segments. (D) View of the tail, showing the post-abdominal respiratory siphon.

Immediately following the first paediatric consultation, the adolescent was instructed to use a clean container for urinary and fecal elimination. Sterilized closed containers were provided by the medical team to collect specimens. Domiciliary samples from both stool and urine were obtained. Upon examination by the local hospital's microbiology team, a specimen of dipterous nematoceran larvae from the *Psychodidae* family, subfamily *Psychodinae*, was identified in a stool sample (Figure 1). Concurrently, a medical entomologist confirmed the identification of *Psychodinae* larvae, consistent with the genus *Clogmia* spp., in a urinary sample.

It was decided to hospitalize the adolescent for one week for close monitoring of urinary and fecal elimination in a clean container. During her hospital stay, she was subjected to a trial of oral ivermectin at a dose of 200 mcg/kg/day for two consecutive days. No alterations in her stools or urine were identified during this period. Physical examination was unremarkable, and the gynecological examination revealed no abnormalities, with no visible signs of inflammation or other types of lesions in the genital, periurethral, and perianal areas.

Since the beginning of the investigation, the parents indicated that the sanitation facilities were adequate and that the water from the plumbing system was sourced from a well, which served as the primary water source throughout the household. The water underwent frequent quality testing, which was confirmed by the medical team. Waste was drained into a private cesspit. It is noteworthy that the five other residents of the household reported no symptoms.

After identifying the larvae and explaining the fly's life cycle to the adolescent and her parents, along with providing illustrative images, they recognized the presence of a similar fly near the toilet, something they had not noticed before. Following this finding, a complete renovation of the sanitation facilities was carried out, and the drainage system was isolated. After discharge from the hospital, the patient continued to eliminate dead larvae, though in reduced quantities, for an additional two months. However, after those interventions in the house finished, larval elimination ceased entirely, with no recurrence observed.

Simultaneously, during the investigation, parasitological examinations of the stools were conducted at two distinct times, with three separate stool samples collected on alternate days, and all results revealed no abnormalities. Laboratory tests, including urinalysis, were all normal, except for an IgA deficiency. Abdominal and pelvic ultrasounds revealed no alterations. At the insistence of her parents, she also underwent a colonoscopy in a private setting, with normal results.

## Discussion

Some mechanisms for pseudomyiasis have been proposed, including the ingestion of fly larvae in contaminated food or water, the colonization of urinary catheters, or, although rare, the laying of eggs on mucosal surfaces such as the anal or genital areas, with larvae subsequently traveling towards the rectum or urogenital canal [2,3]. Another explanation involves eggs deposited on freshly collected stool samples exposed to the environment, which can hatch into visible larvae within hours, creating the impression that they were passed in the stool [2].

A four-stage life cycle has been described for *C. albipunctata*, consisting of egg, larva (four instars), pupa, and adult fly, with a duration reported to be at least 17 days, with eggs hatching within 32-48 hours [3] and larval development typically requiring between 12 and 15 days [2]. Some authors suggest a longer full cycle period of 27 ± 5 days, depending on temperature and availability of nutrients [3]. Larvae have specific breathing requirements, as they are not adapted to survive for extended periods in oxygen-poor environments without access to atmospheric air [2-4]. Considering these biological characteristics and the time required for larval development, it is unlikely that they could survive, let alone develop, in the human gastrointestinal or urogenital tracts [2].

In this report, hypothesizing pseudomyiasis as a possible diagnosis and considering the most likely mechanism of fly oviposition near the urethra and anus, especially since some of the larvae were alive, we raise the question, shared by other authors, of how oviposition occurs in these orifices and how the eggs or hatching larvae would move inward without the patient noticing [3,4]. Additionally, if larvae are physically present, whether externally or internally, they may induce local mucosal irritation, even if only temporarily [2]. Such resulting lesions were not observed, nor in the majority of other reported cases [2,3]. Furthermore, the identification of the larvae was based on samples collected from a supposed clean container at the patient’s home, as they were never collected or directly observed in the adolescent by the medical team. Moreover, imaging and laboratory investigations revealed no significant abnormalities.

In conclusion, we agree that a definitive diagnosis should not rely only on the patient's report. It is essential to directly observe the suspected causative agent, document it through imaging, and, when available, ensure more precise identification of the larvae using advanced techniques such as electron microscopy or molecular biology, in order to eliminate any doubt and avoid unnecessary treatment [2-4].

Consequently, definitive conclusions cannot be drawn as to whether these instances represent pseudomyiasis or are merely incidental findings in the environment. Nevertheless, the evidence leans towards the latter explanation. Another possibility is delusional parasitosis involving environmental larvae [3], despite the absence of other psychiatric manifestations in the adolescent.

The key message is to focus on eliminating the source of the problem after identifying the implicated larval species. In this case, this was the turning point, leading to the eradication of this nuisance pest.

## Conclusions

In conclusion, while *Psychodinae* flies (drain flies) have been increasingly associated with urogenital and intestinal myiasis, their larvae are typically not capable of surviving in these tracts. Consequently, it is likely that most reports, including this case, represent incidental findings or, in rare instances, may indicate pseudomyiasis rather than true myiasis.

A definitive diagnosis should not rely only on the patient’s report. The larvae and any associated lesions must be directly observed in the patient, accurately identified, and supported by imaging to avoid doubt. Furthermore, identifying and eliminating the source of infestation is crucial to eradicating the problem and preventing its recurrence.
